# Impact of Elevated Lipoprotein A on Clinical Outcomes in Patients Undergoing Percutaneous Coronary Intervention: A Systematic Review and Meta-analysis

**DOI:** 10.7759/cureus.61069

**Published:** 2024-05-25

**Authors:** Tanya Sinha, Manisha Guntha, Abshiro H Mayow, Aung K Zin, Sandipkumar S Chaudhari, Muhammad Waqas Khan, Samer Kholoki, Areeba Khan

**Affiliations:** 1 Internal Medicine, Tribhuvan University, Kathmandu, NPL; 2 Internal Medicine, Sinai-Grace Hospital, Detroit, USA; 3 School of Medicine, St. George’s University, Chicago, USA; 4 Internal Medicine, University of Medicine, Mandalay, Mandalay, MMR; 5 Cardiothoracic Surgery, University of Alabama at Birmingham, Birmingham, USA; 6 Family Medicine, University of North Dakota School of Medicine and Health Sciences, Fargo, USA; 7 Medicine, Services Institute of Medical Sciences, Lahore, PAK; 8 Internal Medicine, La Grange Memorial Hospital, Chicago, USA; 9 Critical Care Medicine, United Medical and Dental College, Karachi, PAK

**Keywords:** systematic review and meta-analysis, mortality, percutaneous coronary intervention, cardiovascular outcomes, lipoprotein (a)

## Abstract

Lipoprotein(a) (Lp(a)) is an inherited lipoprotein particle associated with increased risk of atherosclerotic cardiovascular (CV) diseases. However, its impact on outcomes after percutaneous coronary intervention (PCI) remains unclear. The objective of this study was to assess the relationship between elevated Lp(a) levels and major adverse cardiovascular events (MACEs) and other outcomes in patients undergoing PCI. We systematically searched Embase, MEDLINE/PubMed, and Web of Science for studies published from 2015 to 2024 comparing CV outcomes between patients with elevated versus non-elevated Lp(a) levels after PCI. Primary outcome was MACE. Secondary outcomes included all-cause mortality, CV mortality, stroke, myocardial infarction, and revascularization. Risk ratios (RRs) were pooled using a random-effect model. Fifteen studies with 45,059 patients were included. Patients with elevated Lp(a) had a significantly higher risk of MACE (RR 1.38, 95% confidence interval (CI) 1.23-1.56). Elevated Lp(a) was also associated with increased risks of all-cause death (RR 1.26), CV death (RR 1.58), myocardial infarction (RR 1.44), revascularization (RR 1.38), and stroke (RR 1.18). Heterogeneity was considerable for some outcomes. This meta-analysis demonstrates that elevated Lp(a) levels are associated with worse CV outcomes, including higher rates of MACE, mortality, and recurrent ischemic events in patients undergoing PCI. Novel therapeutic approaches specifically targeting Lp(a) reduction may help mitigate residual CV risk in this high-risk population.

## Introduction and background

The advent of percutaneous coronary intervention (PCI) has transformed the treatment landscape for coronary artery disease (CAD), providing a less invasive method to reestablish blood flow to the oxygen-deprived heart muscle [1]. Despite notable progress in procedural techniques, medication adjuncts, and device innovations, many patients still confront recurring ischemic episodes and vessel narrowing post-PCI [2]. This highlights the imperative to recognize and manage remaining risk factors that could influence less than optimal long-range results.

Lipoprotein(a) (Lp(a)) stands as an inherited lipoprotein with atherosclerotic tendencies, standing alone as a risk element for atherosclerotic cardiovascular (CV) disease, vascular thrombosis, and calcific aortic stenosis [3-4]. Its occurrence in the elevated range encompasses about 20% of subjects [5]. Structurally akin to low-density lipoprotein (LDL), Lp(a) comprises cholesterol and is tethered to apolipoprotein(a) and apolipoprotein B100. Notably, it shares similarities with plasminogen structurally, and the most atherogenic subtype bears over a 2.5-fold risk for coronary heart disease [6]. Substantial epidemiological, meta-analytic, Mendelian randomization, and genome-wide association research has pinpointed elevated Lp(a) as an autonomous risk factor, conceivably causal, for atherosclerotic CV disease [7-8]. 

However, the substantial and expanding body of evidence primarily derives from investigations involving the general populace lacking established CV diseases. Discordant findings persist regarding the impact of heightened Lp(a) levels in secondary prevention [9]. Over recent years, mounting data consistently indicate the potential reduction of Lp(a) through PCSK9 inhibitor therapy [10-11]. Consequently, Lp(a) is gaining acknowledgment as a modifiable CV risk element, prompting speculation that diminishing Lp(a) could further mitigate residual CV risk. 

Understanding the role of Lp(a) in this high-risk population is of paramount importance, as it may guide personalized treatment strategies, including more intensive medical therapy, closer follow-up monitoring, or alternative revascularization approaches. In addition, the identification of Lp(a) as a modifiable risk factor may pave the way for the development of novel therapeutic interventions specifically targeting this unique lipoprotein particle. This review aims to provide a comprehensive analysis of the current evidence regarding the impact of Lp(a) on CV outcomes in patients undergoing PCI. By elucidating the mechanisms underlying the detrimental effects of Lp(a) in this high-risk population, we hope to contribute to the development of more effective strategies for risk stratification, prevention, and management of ischemic complications following PCI.

## Review

Methodology

This study was performed and reported as per the guidance of the Preferred Reporting Items for Systematic Reviews and Meta-Analyses (PRISMA) guidelines. 

Search Strategy and Study Selection

Embase, MEDLINE/PubMed, and Web of Science were searched for records published from January 1, 2015 to April 15, 2024. We confined the search to articles in English. In addition, a bibliographic search of pertinent reviews was conducted along with eligible studies to uncover further publications. The keywords used to search for relevant articles included "lipoprotein (a)," "percutaneous intervention," and "major adverse cardiovascular events" along with their synonyms and medical subject heading (MeSH) terms. The search was done by two investigators independently. Disagreements between two authors were resolved through discussion. Briefly, we included studies if they had (a) individuals undergoing PCI and (b) reported levels of Lp(a) on the nominal scale (with two levels including high and low). We excluded studies that assessed the relationship of Lp(a) as continuous variable with CV outcomes. We also excluded non-original studies including reviews, case series, editorials, and case reports.

Two independent reviewers conducted screening of titles and abstracts. Discrepancies were resolved through consensus. Full-text publications underwent screening, and those meeting the eligibility criteria were chosen for the extraction of data. One reviewer conducted data extraction, with a second reviewer performing quality checks of extracted data. Discrepancies were reconciled by the third reviewer. 

Outcomes and Risk-of-Bias Assessment 

Primary outcome assessed in this meta-analysis included major cardiovascular adverse events (MACEs). For secondary outcomes, we assessed all-cause mortality, CV mortality, stroke, myocardial infarction, and revascularization.

Assessment of risk of bias of included studies was done using the Newcastle-Ottawa Scale (NOS), which is a validated tool for assessing the bias assessment for observational studies. It assesses three important domains: the study group selection, group comparison, and the assessment of exposure/outcome. In our review, studies were considered high quality if they received a score of 7 or above on the NOS. Two independent reviewers assessed the risk of bias of each included study using the NOS. Any discrepancies were resolved through discussion.

Statistical Analysis

For the statistical analysis, we used Review Manager (RevMan) version 5.4.1 (The Cochrane Collaboration, available at revman.cochrane.org). To compare the risk of outcomes between two groups, pooled risk ratio (RR) was computed with their 95% confidence interval (CI). A cut-off of the p-value was kept at 0.05. Heterogeneity among the study results was presented as I-square. An I-square value of 50% or more shows significant heterogeneity. We used the random-effect model irrespective of the heterogeneity among the study results due to variations in the study population and study design.

Results

**Figure 1:**
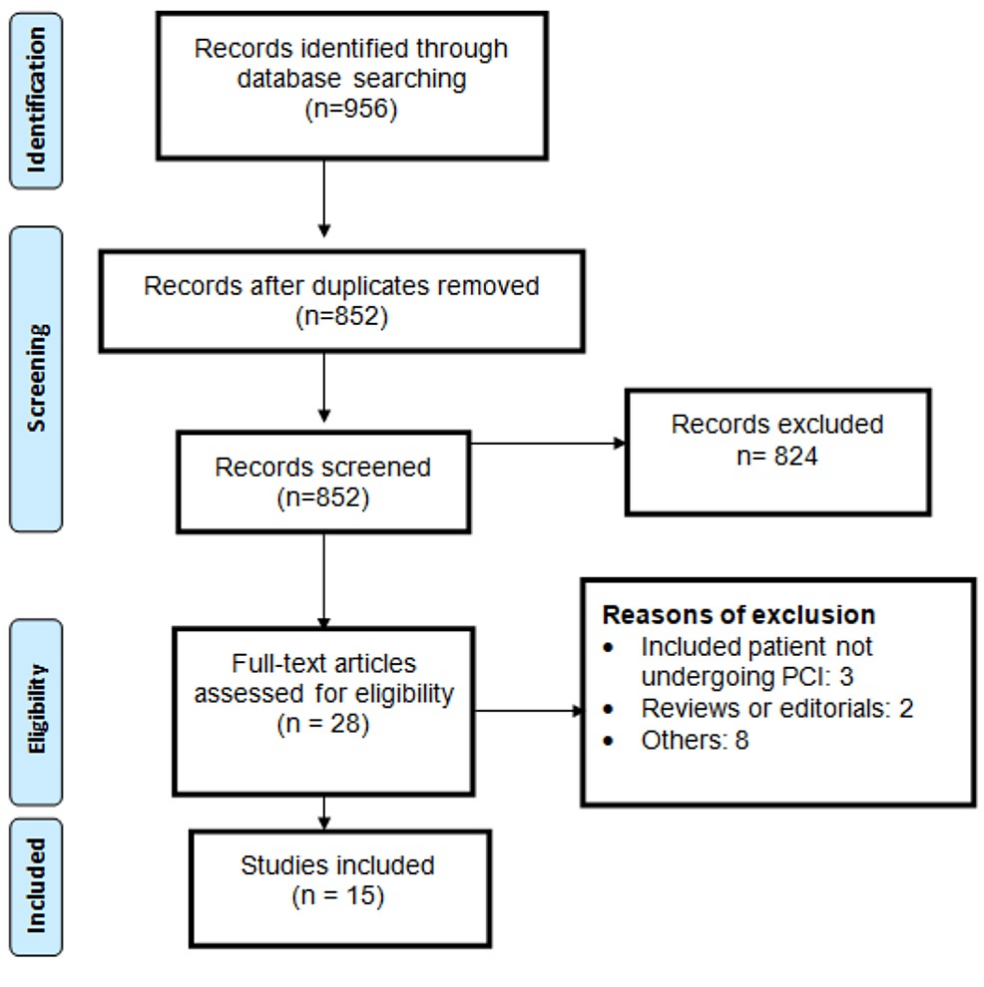
PRISMA flowchart of study selection PRISMA: Preferred Reporting Items for Systematic reviews and Meta-Analyses

We identified 956 articles through online database searching (PubMed: 466, Web of Science: 338, EMBASE: 152). In the initial screening, 28 studies were found eligible for detailed assessment based on eligibility criteria. Finally, 15 studies were included in the meta-analysis. Figure 1 shows the process of study selection. Table 1 presents the characteristics of included studies. The pooled sample size of this meta-analysis was 45,059, including 15,534 in the elevated Lp(a) group and 29,515 in the non-elevated Lp(a) group. Majority of the studies were conducted in China, followed by Japan and Korea. Table 2 presents the quality assessment of the included studies. Majority of the studies were having high quality in terms of selection, comparison of group, and assessment of exposure and outcomes. 

**Table 1:** Characteristics of the included studies NR: not reported

Author name	Year	Region	Definition of elevated	Follow-up	Groups	
					Elevated	Non-elevated
Cui et al. [12]	2022	China	Lp(a) >30 mg/dl	2.4 years	3495	6564
Dykun et al. [13]	2022	Spain	NR	3.1 years	1817	3124
Hishikari et al. [14]	2020	Japan	Lp(a) >40 mg/dL	2 years	113	297
Kimura et al. [15]	2022	Japan	Lp(a) >30 mg/dl	3 years	109	386
Konishi et al. [16]	2016	Japan	NR	4.7 years	454	450
Liu et al. [17]	2019	China	NR	1 year	176	174
Liu et al. [18]	2020	China	Lp(a) >30 mg/dl	4.9 years	1247	2831
Park et al. [19]	2015	Korea	Lp(a) >50 mg/dl	3 years	94	413
Sami et al. [20]	2023	Egypt	Lp(a) >30 mg/dl	In-hospital	21	49
Wu et al. [21]	2020	China	Lp(a) >30 mg/dl	In-hospital	726	566
Xu et al. [22]	2018	Japan	NR	2 years	213	214
Yang et al. [23]	2022	China	Lp(a) >30 mg/dl	2 years	203	562
Yoon et al. [24]	2021	Korea	Lp(a) >30 mg/dl	7.4 years	3747	8317
Zhang et al. [25]	2023	China	Lp(a) >30 mg/dl	3 years	834	1252
Zhu et al. [26]	2020	China	Lp(a) >30 mg/dl	2 years	2285	4316

**Table 2:** Risk-of-bias assessment of the included studies

Author name	Selection	Comparability	Ascertainment	Overall
Cui et al. [12]	3	2	3	Good
Dykun et al. [13]	2	1	2	Fair
Hishikari et al. [14]	3	2	3	Good
Kimura et al. [15]	3	3	3	Good
Konishi et al. [16]	3	2	2	Good
Liu et al. [17]	3	2	2	Good
Liu et al. [18]	4	2	3	Good
Park et al. [19]	3	2	2	Good
Sami et al. [20]	4	2	2	Good
Wu et al. [21]	3	2	2	Good
Xu et al. [22]	3	1	2	Fair
Yang et al. [23]	4	2	2	Good
Yoon et al. [24]	3	3	2	Good
Zhang et al. [25]	3	2	3	Good
Zhu et al. [26]	4	2	3	Good

Meta-analysis of the Outcomes

**Figure 2:**
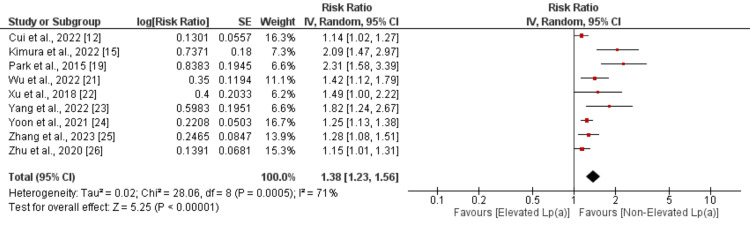
Effect of elevated Lp(a) on MACE References: [12,15,19,21-26]

MACE: To evaluate the impact of elevated Lp(a) on the risk of MACE, nine studies underwent pooled analysis, with results depicted in Figure 2. The analysis revealed a significantly heightened risk of MACE in patients with elevated Lp(a) compared to those with non-elevated levels (RR: 1.38, 95% CI: 1.23 to 1.56, p-value < 0.01). Considerable heterogeneity was observed among the study outcomes (I-square: 71%). All studies addressing this outcome reported an increased risk of MACE in patients with elevated Lp(a), with statistically significant findings. Sensitivity analysis, excluding studies using a cutoff for elevated Lp(a) other than 30 mg/dL, was conducted. The pooled analysis of seven studies utilizing a cutoff of 30 mg/dL demonstrated a significantly elevated risk of MACE in patients with elevated Lp(a) (RR: 1.30, 95% CI: 1.17 to 1.45).

Secondary outcomes: Table 3 depicts the association between elevated Lp(a) levels and various clinical outcomes. RRs above 1 indicate an increased risk of adverse events in patients with elevated Lp(a). Elevated Lp(a) is significantly associated with a higher risk of all-cause death (RR 1.26), CV death (RR 1.58), myocardial infarction (RR 1.44), revascularization (RR 1.38), and stroke (RR 1.18). Variability in the strength of association was observed across outcomes, with higher heterogeneity noted in revascularization (I-square 73%) and myocardial infarction (I-square 42%) compared to other outcomes. These findings underscore the potential clinical importance of monitoring and managing elevated Lp(a) levels to mitigate CV risks. 

**Table 3:** Effect of elevated Lp(a) on secondary outcomes RR: risk ratio; CI: confidence interval

Outcomes	RR (95% CI)	I-square
All-cause death	1.26 (1.17 to 1.36)	0%
Cardiovascular death	1.58 (1.31 to 1.91)	28%
Myocardial infarction	1.44 (1.20 to 1.72)	42%
Revascualarization	1.38 (1.19 to 1.60)	73%
Stroke	1.18 (1.00 to 1.39)	0%

Discussion

An alarming statistic reveals that one-fifth of the global population, approximately 1.4 billion individuals, carries elevated lipoprotein(a) (Lp(a)) levels of 50 mg/dL or higher (≥125 nmol/L), predisposing them to a heightened risk of atherosclerotic cardiovascular disease (ASCVD) [27,28]. This meta-analysis offers a comprehensive overview of available evidence concerning the adverse outcomes linked to elevated Lp(a) levels in patients undergoing PCI. The analysis underscores that elevated Lp(a) levels are correlated with an increased risk of MACEs, all-cause mortality, and CV mortality.

Prior extensive epidemiological and genetic investigations involving the general populace devoid of established CAD have revealed that Lp(a) levels are genetically predisposed and may serve as a causative element in the onset of atherosclerotic CV disease [6,8]. Elevated Lp(a) levels exhibit a dose-dependent escalation in the likelihood of CV events [29]. Furthermore, supplementing the conventional risk assessment model with Lp(a) enhances the prediction of CV risk [30]. 

In all investigations comparing MACE risk between individuals with elevated and non-elevated Lp(a), a consistent finding emerged: patients with elevated Lp(a) faced significantly increased MACE risk compared to their counterparts. Nevertheless, heterogeneity across study outcomes was notable. This variation may be attributed to diverse factors, such as variations in sample size, duration of follow-up, baseline risk profiles, and ethnic backgrounds.

Interestingly, the CV risk linked to Lp(a) persists regardless of LDL-C levels [6]. The past study reported that the occurrence rate of major adverse CV events associated with increased Lp(a) levels remained consistent despite variations in LDL-C levels [31]. While therapies targeting high LDL-C levels are currently available to mitigate the CV disease risk, there are no approved treatments specifically designed to reduce levels of Lp(a) and therefore diminish residual CV disease risk. However, in Germany, progressive CV disease with Lp(a)-hyperlipoproteinemia has been recognized as a distinct indication for apheresis in the guidelines of statutory health insurance funds [32]. Moreover, Australian integrated guidelines aimed at enhancing the care of patients with familial hypercholesterolemia (FH) suffering from ASCVD and elevated Lp(a) levels (≥150 nmol/L) have recommended apheresis to attenuate ASCVD progression in individuals who cannot achieve LDL-C targets despite receiving maximally tolerated drug therapy [33].

The European Atherosclerosis Society advocated for an optimal level of Lp(a) of <50 mg/dL [7]. However, emerging evidence indicates that poor CV outcome risk increased with increase in Lp(a) levels of 24 to 30 mg/dL in the general population [34-35]. Furthermore, there was a more notable enhancement in predicting CV events when employing the Lp(a) cut-off of 30 mg/dL [36]. A recent review corroborated that CV adverse event risk significantly escalates at Lp(a) levels >30 mg/dL [37]. Given that most studies in this meta-analysis used a cutoff of <30 mg/dL, it suggests that this levels of Lp(a) needs to be used to assess individuals undergoing PCI. However, further studies are needed assessing the sensitivity, specificity, and other diagnostic parameters of developing poor CV outcomes.

At present, available treatments can decrease Lp(a) levels by approximately 20-30 percent, yet the CV advantages of lowering Lp(a) remain unproven [38]. However, there is a prospective pharmacological intervention in development capable of reducing Lp(a) levels by up to 90% [5]. Patients who have undergone PCI and received optimal medical management afterward remain at heightened risk of recurrent ischemic events, positioning them as primary candidates for such therapy. In addition, our findings from PCI patients could inform the designing and execution of future clinical trials exploring whether reducing levels of Lp(a) can diminish CV events.

Study Limitations

The current meta-analysis is subject to certain limitations. First, all included studies were observational, which could introduce selection bias. Second, the assessment of various significant variables and outcomes, such as compliance with antiplatelet agents during follow-up, familial history of premature atherosclerotic CV events, and stent thrombosis, was hindered by insufficient data in individual studies. Another notable limitation of the meta-analysis is the potential variability and incomplete understanding of dyslipidemia treatment regimens across the included studies. Dyslipidemia, characterized by abnormal lipid levels in the blood, is a significant risk factor for CV diseases, and its effective management plays a crucial role in mitigating adverse outcomes. However, the individual studies may have employed different therapeutic approaches or lacked comprehensive documentation of the specific treatments used for managing dyslipidemia in their respective study populations.

## Conclusions

Based on the findings of this meta-analysis, elevated Lp(a) levels are associated with an increased risk of MACEs, all-cause mortality, and CV mortality in patients undergoing PCI. While current treatments can modestly reduce Lp(a) levels, novel therapies capable of substantially lowering Lp(a) could potentially mitigate the residual CV risk in this high-risk population. Further research is warranted to determine the optimal Lp(a) level for risk stratification and to explore the potential benefits of targeted Lp(a) reduction in reducing CV events in patients undergoing PCI.
